# The Biological Effects of Compound Microwave Exposure with 2.8 GHz and 9.3 GHz on Immune System: Transcriptomic and Proteomic Analysis

**DOI:** 10.3390/cells11233849

**Published:** 2022-11-30

**Authors:** Chuanfu Yao, Hui Wang, Liu Sun, Ke Ren, Ji Dong, Haoyu Wang, Jing Zhang, Xinping Xu, Binwei Yao, Hongmei Zhou, Li Zhao, Ruiyun Peng

**Affiliations:** Beijing Institute of Radiation Medicine, Beijing 100850, China

**Keywords:** microwave, immune system, effects, transcriptomic, proteomic

## Abstract

It is well-known that microwaves produce both thermal and nonthermal effects. Microwave ablation can produce thermal effects to activate the body’s immune system and has been widely used in cancer therapy. However, the nonthermal effects of microwaves on the immune system are still largely unexplored. In the present study, we exposed rats to multifrequency microwaves of 2.8 GHz and 9.3 GHz with an average power density of 10 mW/cm^2^, which are widely used in our daily life, to investigate the biological effects on the immune system and its potential mechanisms. Both single-frequency microwaves and multifrequency microwaves caused obvious pathological alterations in the thymus and spleen at seven days after exposure, while multifrequency microwaves produced more pronounced injuries. Unexpectedly, multifrequency microwave exposure increased the number of both leukocytes and lymphocytes in the peripheral blood and upregulated the proportion of B lymphocytes among the total lymphocytes, indicating activation of the immune response. Our data also showed that the cytokines associated with the proliferation and activation of B lymphocytes, including interleukin (IL)-1α, IL-1β and IL-4, were elevated at six hours after exposure, which might contribute to the increase in B lymphocytes at seven days after exposure. Moreover, multifrequency microwave exposure upregulated the mRNA and protein expression of B cell activation-associated genes in peripheral blood. In addition to immune-associated genes, multifrequency microwaves mainly affected the expression of genes related to DNA duplication, cellular metabolism and signal transduction in the peripheral blood and spleen. In conclusion, multifrequency microwaves with 2.8 GHz and 9.3 GHz caused reversible injuries of the thymus and spleen but activated immune cells in the peripheral blood by upregulating mRNA and protein expression, as well as cytokine release. These results not only uncovered the biological effects of multifrequency microwave on the immune system, but also provide critical clues to explore the potential mechanisms.

## 1. Introduction

With the rapid development of microwave technologies, the biological effects induced by microwaves have attracted increasing attention. It has been reported that microwaves can cause multiple organ symptoms, called “electromagnetic hypersensitivity (EHS)”. Moreover, the nervous system, cardiovascular system, immune and hematologic systems, and skin are frequently affected [1]. The biological effects of microwaves with different frequencies, power densities, and duration periods have been widely investigated in the nervous systems [2,3,4]. Moreover, several potential mechanisms were also demonstrated both in vivo and in vitro [5,6]. For example, 2.8 GHz microwave exposure induced autophagy of neurons through the miR-30a-5p/AMPKα2 signaling pathway [7]. The immune system plays critical roles in protecting humans against both internal mutations and external stimuli. However, microwave-induced biological effects on the immune system are still largely unexplored. 

It is well known that microwaves can generate thermal and nonthermal effects on the immune system. Microwave ablation, which mainly relies on the thermal effects of microwaves, has emerged as an effective adjuvant approach for cancer therapy [8,9,10]. It has been reported that microwave ablation can boost obvious immune responses, such as inducing T-helper (Th) 1 immune response and activating natural killer cells [11]. A few groups also reported the nonthermal effects of microwaves on the immune system, which focused on the alteration of immune cell proliferation, phagocytosis, and the production of antibodies [12,13]. However, controversial results have also been reported, which are closely associated with the frequency, power density, and duration of exposure [14,15]. 

In this study, it was shown that 2.8 GHz and 9.3 GHz microwave irradiation caused structural injuries in the thymus and spleen. Furthermore, a multifrequency microwave of 2.8 GHz and 9.3 GHz produced more impressive injuries. Interestingly, B lymphocytes were upregulated by multifrequency microwaves, which might be attributed to the activation of compensatory mechanisms. Moreover, the transcriptome and proteome results suggested that alterations in the mRNA and protein expression profiles in the peripheral blood and spleen, especially cellular metabolism- and immune activation-associated genes, might contribute to immune homeostasis after microwave exposure. 

## 2. Materials and Methods 

### 2.1. Animals

Eighty 6- to 8-week-old adult male Wistar rats (180–220 g) were purchased from Vital River Laboratory Animal Technology Co., Ltd. (Beijing, China). The rats were randomly divided into four groups: Sham group, 2.8 GHz single-frequency microwave-exposed group (S10), 9.3 GHz single-frequency microwave-exposed group (X10), and 2.8 GHz plus 9.3 GHz multifrequency microwaves exposed group (XS10). The animal experiments were approved by the Ethical Committee of Beijing Institute of Radiation Medicine (IACUC-DWZX-2020-781), and all procedures were carried out in compliance with the National Institute of Health Guide for the Care and Use of Laboratory Animals. 

### 2.2. Microwave Exposure

The microwave exposure system and procedures have been described previously [16,17,18]. In this study, rats in the S10 and X10 groups were exposed to 2.8 GHz and 9.3 GHz pulsed microwaves for 6 min with an average power density of 10 mW/cm^2^. The rats in the XS10 group were sequentially exposed to 2.8 GHz microwaves and 9.3 GHz microwaves for 6 min each. Moreover, rats in the Sham group were treated the same as the rats in the microwave-exposed groups, but without microwave exposure.

### 2.3. Histopathological Analysis

Rats from each group were sacrificed for pathological examination at 6 h, 7 d, 14 d and 28 d after microwave exposure. The thymus and spleen were removed and fixed in 10% buffered formalin solution, and then the tissues were embedded in paraffin and cut into 3 μm thick sections in the coronal plane. The sections of the thymus and spleen were then stained with hematoxylin-eosin (H&E) and observed under a light microscope by a pathologist blind to the group assignment (Leica, Wetzlar, Germany). 

### 2.4. Transmission Electron Microscopy (TEM)

At 7 d after exposure, the rats from each group were sacrificed and the spleens were removed. Then, specimens (1 mm^3^) were obtained from the spleen, placed in 2.5% glutaraldehyde and postfixed with 1% osmium tetroxide. After graded ethyl alcohols, the cubes were embedded in EPON618. Thin sections laid on copper mesh were stained with heavy metals, uranyl acetate, and lead citrate. The ultrastructure of the spleen was observed by using a Hitachi-H7650 transmission electron microscope (Hitachi, Tokyo, Japan). 

### 2.5. Hemogram Analysis 

At 6 h, 7 d and 28 d after microwave exposure, rats from each group were anesthetized, and anticoagulant blood samples were collected from the inferior vena cava (IVC). The number of immune cells, including white blood cells, lymphocytes and neutrophils, was analyzed by an automatic blood cell counter (XN1000, Sysmex Europe GmbH, Kobe, Japan).

### 2.6. Flow Cytometry

Peripheral blood anticoagulated with ethylenediamine tetraacetic acid (EDTA) was collected from the IVC of rats at 6 h, 7 d and 28 d after microwave exposure. B and T lymphocytes, two major subtypes of lymphocytes, and CD4^+^ and CD8^+^ T lymphocytes were analyzed by flow cytometry (BD Accuri C6, BD Biosciences, Franklin Lake, NJ, USA). Briefly, anticoagulated peripheral blood was labeled with antibody Panel 1: phycoerythrin (PE)-conjugated mouse anti-rat CD45 antibody, allophycocyanin (APC)-conjugated mouse anti-rat CD3 antibody, fluorescein isothiocyanate (FITC)-conjugated mouse anti-rat CD45RA antibody; or antibody Panel 2: APC-conjugated mouse anti-rat CD3 antibody, FITC-conjugated mouse anti-rat CD4 antibody, PE-conjugated mouse anti-rat CD8a antibody (Biolegend, Santiago, CA, USA), for 30 min at room temperature. Then, the erythrocytes were lysed with 1 × lysis buffer (BD Biosciences, Franklin Lake, NJ, USA) for 8 min at room temperature. After washing 3 times, the subtypes of lymphocytes were analyzed by flow cytometry. 

### 2.7. Multiplex Immunoassay

At 6 h and 7 d after microwave exposure, peripheral blood was collected from the IVC of the rats in each group, and the sera were isolated. The contents of cytokines and chemokines in the serum were analyzed by a Luminex immunoassay kit (Cytokine & Chemokine 22-Plex Rat ProcartaPlex™ Panel, Invitrogen, Karlsbad, CA, USA) according to the manufacturer’s instructions. 

### 2.8. Transcriptome Analysis

At 7 d after exposure, rats from each group were euthanized, and the peripheral blood and spleen were collected (n = 5/group). Then, total RNA was extracted from both peripheral blood and spleen by TRIzol reagent (Invitrogen, Karlsbad, CA, USA). The concentration, quality and integrity of total RNA were detected using a NanoDrop Spectrophotometer (Thermo Fisher Scientific, Waltham, MA, USA). 

RNA sequencing was performed using the NovaSeq 6000 platform (Illumina). Reference genome and gene model annotation files were downloaded from the genome website. The filtered reads were mapped to the reference genome using HISAT2 v2.0.5. HTSeq (0.9.1) statistics were used to compare the read count values on each gene as the original expression of the gene, and then FPKM were used to standardize the expression. Then, the differential expression of genes was analyzed by DESeq2 with the following screening conditions: expression difference multiple |log_2_FoldChange| > 1, significant *q*-value ≤ 0.05. In the meantime, the R language Pheatmap (1.0.8) software package was used to perform clustering analysis of all differentially expressed genes in the samples. 

All the genes were mapped to terms in the Gene Ontology (GO) database, and the numbers of differentially enriched genes in each term were calculated. TopGO was used to perform GO enrichment analysis on the differentially expressed genes (DEGs), calculate the *p* value by hypergeometric distribution method (the standard of significant enrichment is *p* value < 0.05), and find the GO term with significantly enriched DEGs to determine the main biological functions performed by DEGs. The enrichment analysis of the KEGG pathway of DEGs focused on the significant enrichment pathway with *p*-value < 0.05.

### 2.9. Proteomics Analysis

The blood and spleen were collected at 7 d after exposure as described above. Briefly, the proteins were extracted, digested, desalinated, and then fractionated using a C18 column (Waters, Milford, MA, USA) on a Rigol L3000 high-performance liquid chromatograph (HPLC) operating at 0.7 mL/min (Rigol, Suzhou, China). For spectral library generation, samples were fractionated using a high pH reversed-phase fractionator and measured in data dependent acquisition (DDA) mode. And then, the protein expression was analyzed by data-independent acquisition (DIA), which consisted of one MS1 scan (350 to 1350 *m*/*z*, resolution 120,000 maximum injection time 50 ms, AGC target 4E5) and 60 segments at varying isolation windows from 349 *m*/*z* to 1500 *m*/*z*. The stepped normalized collision energy was 35. Differentially expressed proteins (DEPs) were analyzed as previously reported [19]. 

GO and InterPro (IPR) analyses were conducted using the interproscan-5 program against the nonredundant protein database and the COG (Clusters of Orthologous Groups) database. KEGG was used to analyze the protein families and pathways. The enrichment pipeline was used to perform GO and KEGG enrichment analyses.

### 2.10. Quantitative Reverse Transcription Polymerase Chain Reaction (qRT-PCR)

Total RNA was extracted from peripheral blood and spleen by a standard column purification method using an RNAeasy Mini Kit (Qiagen, Düsseldorf, Germany) according to the manufacturer’s instructions. Then, cDNA was synthesized using an Evo M-MLV RT kit (Accurate Biotechnology, Changsha, China) following the manufacturer’s instructions. The mRNA expression of *Ahcy*, *Atic*, *Dnaja1*, *Il17ra*, *Kiaa0408L*, *Mal*, *Nxf1*, *Pglyrp1*, *Sept1*, *Sowahb*, *Was* and *Zbtb16*, was measured by qRT-PCR using SYBRR Green Realtime Master Mix (TOYOBO, Osaka, Japan) on a Roche Light Cycle 480II (Roche, Basel, Switzerland) following the manufacturer’s instructions. All data were normalized to the expression of glyceraldehyde-3-phosphate dehydrogenase (GAPDH), and the relative mRNA expression was calculated using the 2^−ΔΔCT^ method. The primers for qPCR are listed in Table S1. 

### 2.11. Mass Spectrometry Analysis

The blood and spleen were collected from each group at 7 d after exposure. Protein samples for mass spectrometry analysis were prepared as described above. Briefly, proteins were extracted, and trypsin digested. The samples were analyzed using a U3000 UHPLC system (Thermo Fisher) coupled with an Orbitrap Fusion Mass Spectrometer (Thermo Fisher, Waltham, MA, USA) operating in data-dependent acquisition (DDA) mode. The Orbitrap Fusion Mass Spectrometer was operated in the data-dependent acquisition mode using Xcalibur3.0 software, and there was a single full-scan mass spectrum in the Orbitrap (250–1450 *m*/*z*, 120,000 resolution) followed by 3 seconds of data-dependent MS/MS scans in an Ion Routing Multipole at 30% normalized collision energy (HCD). The resulting MS data were processed using Skyline (v.3.6). The expression of DEPs, including Camk1, Cast, Cbr3, Fth1, Napsa, Stk26, andTimm8b, was detected.

### 2.12. Statistical Analysis

All data are expressed as the mean ± s.e.m. and were analyzed by GraphPad Prism software version 6.0 (GraphPad software, San Diego, CA, USA). Longitudinal data were analyzed by a two-way ANOVA, followed by post hoc testing using the S-N-K method. Differences were considered to be significant if *p* < 0.05.

## 3. Results

### 3.1. Cumulative Pathological Injuries Induced by Microwaves of 2.8 and 9.3 GHz on Immune Organs

**Figure 1:**
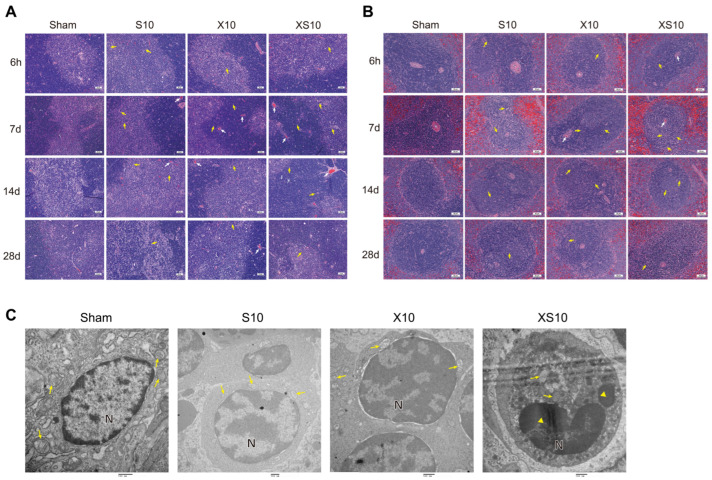
Exposure to multifrequency microwaves of 2.8 GHz and 9.3 GHz caused histopathological injuries to immune organs. Six- to eight-week-old adult male Wistar rats were exposed to 2.8 GHz microwaves (S10), 9.3 GHz microwaves (X10), or 2.8 GHz plus 9.3 GHz microwaves (XS10) at an average power density of 10 mW/cm^2^ for 6 min. At 6 h, 7 d, 14 d and 28 d after exposure, rats in the Sham group, S10 group, X10 group and XS10 group were euthanized, and the immune organs, including the thymus and spleen, were removed. The tissues were processed and stained with hematoxylin-eosin (H&E), and pathological alterations in the thymus, spleen and bone marrow were observed. Representative images of thymus (**A**) and spleen (**B**) at indicated time points after exposure are shown (Scale bar =50 μm). Moreover, the ultrastructural injuries in the spleen were analyzed by transmission electron microscopy (TEM) at 7 d after exposure ((**C**), Scale bar = 500 nm). Congestion was indicated by white arrow, while nuclear fragmentation of lymphocytes was indicated by yellow arrow in (**A**,**B**); Mitochondria are indicated by yellow arrows, and nuclear fragmentation are indicated by yellow triangles in (**C**).

At the indicated time points after microwave exposure, pathological alterations of the immune organs were detected. No obvious changes could be observed in the bone marrow from 6 h to 28 d after microwave exposure (Figure S1). Although no significant alterations were detected at 6 h after exposure, both single-frequency microwaves and multifrequency microwaves caused significant injuries to the thymus and spleen, such as congestion and nuclear fragmentation of the lymphocytes, at 7 d and 14 d after exposure. However, the structure of the thymus and spleen had almost recovered to normal by 28 d after exposure, which might be due to self-recovery mechanisms. Importantly, exposure to multifrequency microwaves produced much more impressive injuries at 7 d after exposure (Figure 1A,B). 

We further analyzed the effects of microwave exposure on the ultrastructure of the spleen at 7 d after exposure. Intact cell structure, orderly arranged chromatin, and well-distributed mitochondria with transversely and longitudinally arranged cristae were observed in the Sham group. Only slight alterations could be detected in the spleen of single-frequency microwave-exposed groups. However, exposure to multifrequency microwaves resulted in condensation of the lymphocyte nuclear chromatin and coagulation of the cytoplasmic organelles (Figure 1C). These data suggested that exposure to multifrequency microwaves of 2.8 GHz and 9.3 GHz produced accumulative injuries to the structure of the thymus and spleen.

### 3.2. Multi-Frequency Microwaves Disturbed the Immune Balance in Peripheral Blood

**Figure 2:**
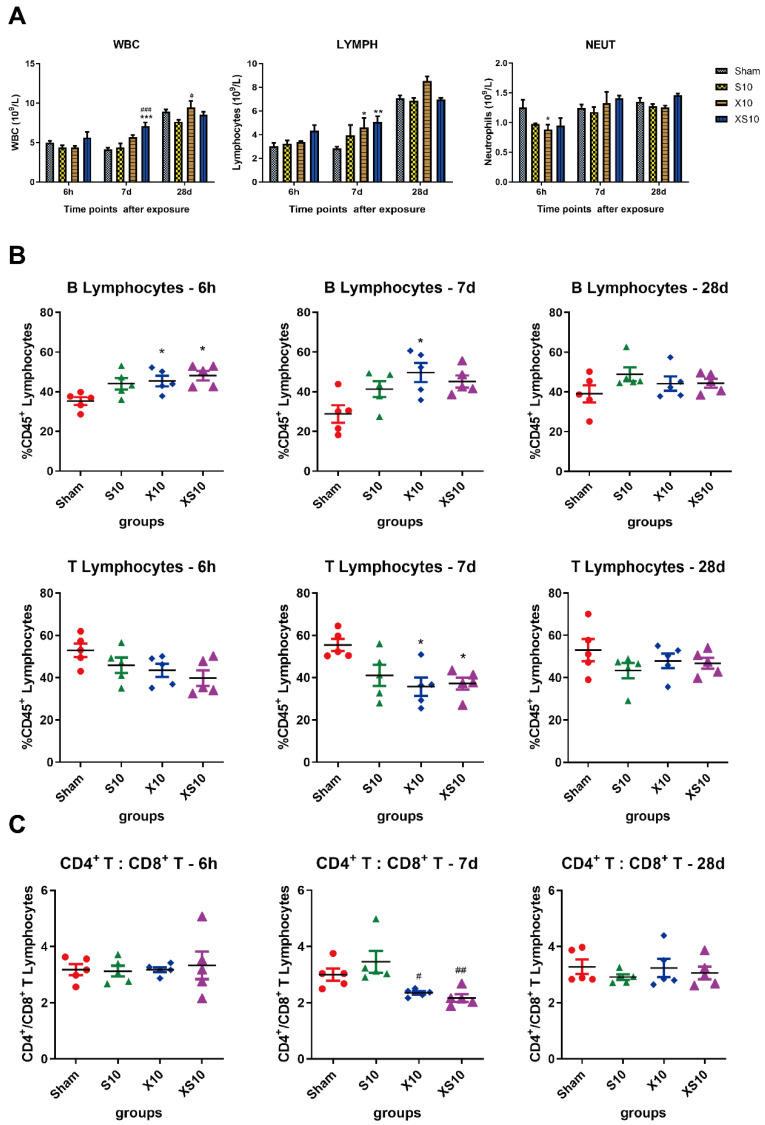
Microwaves regulated the balance of immune cells in the peripheral blood. At 6 h, 7 d and 28 d after microwave exposure, rats from the Sham, S10, X10 and XS10 groups were anesthetized, and anticoagulated blood samples were collected from the inferior vena cava (IVC). The white blood cells (WBC), lymphocytes (LYMPH) and neutrophils (NEUT) were counted (**A**). The immune phenotypes of the lymphocytes were analyzed by flow cytometry, and the percentage of T and B lymphocytes among the total lymphocytes was calculated (**B**). Moreover, the subtypes of T lymphocytes, including CD4^+^ and CD8^+^ T lymphocytes were also detected by flow cytometry, and the proportion of total T lymphocytes was calculated (**C**). Data are presented as mean±s.e.m.; *, *p* < 0.05, **, *p* < 0.01, ***, *p* < 0.001 vs. Sham group; #, *p* < 0.05, ##, *p* < 0.01, ###, *p* < 0.001 vs. S10 group.

Immune homeostasis is important to protect the body from both viral infection and cell mutation. In this study, we analyzed white blood cells, lymphocytes and neutrophils in the peripheral blood after microwave exposure. Generally, multifrequency microwaves, but not single-frequency microwaves, significantly increased the white blood cells and lymphocytes at 7 d after radiation (*p* < 0.001). The number of white blood cells and lymphocytes restored to normal levels at 28 d after exposure, which suggested that the immune balance was transiently regulated by microwaves to maintain immune homeostasis (Figure 2A).

Next, we examined the subtypes of lymphocytes, including T cells and B cells, in the peripheral blood. The proportions of B lymphocytes in the microwave-exposed groups were increased at 6 h and 7 d after exposure, and a much more impressive elevation was detected in the XS10 group (*p* < 0.05), indicating that multifrequency microwave exposure could promote the proliferation of B lymphocytes. As expected, the proportions of T lymphocytes were decreased after microwave exposure. Significant differences were detected between XS10 group and Sham group, as well as between the X10 group and Sham group, at 7 d after exposure (*p* < 0.05). Consistent with the alteration of immune cells in periphery, both the proportions of B lymphocytes and T lymphocytes had recovered to normal levels at 28 d after exposure (Figure 2B). 

Then, the ratio of CD4^+^ T lymphocytes to CD8^+^ T lymphocytes (CD4^+^/CD8^+^ T cells) was analyzed by flow cytometry. It was found that the ratio of CD4^+^/CD8^+^ T cells was obviously downregulated in the X10 and XS10 groups at 7 d after exposure (*p* < 0.05), which was restored to normal at 28 d, suggesting that the balance of T lymphocytes was modulated (Figure 2C). 

### 3.3. Multifrequency Microwaves Transiently Upregulate Cytokines Associated with the Proliferation and Maturation of B Lymphocytes

**Figure 3:**
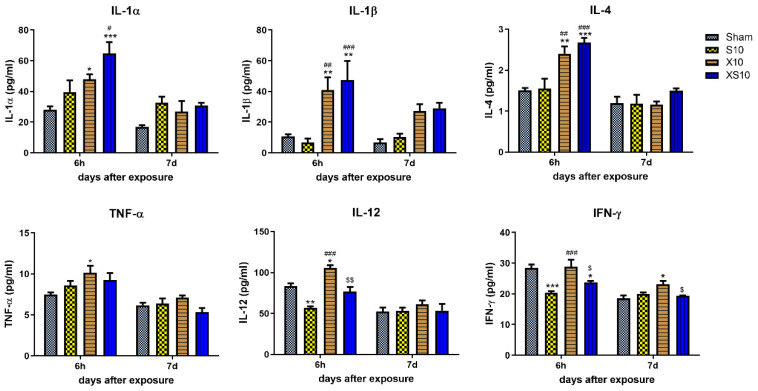
Microwave exposure regulated cytokine levels in peripheral blood. At 6 h and 7 d after microwave exposure, peripheral blood was collected from rats in the Sham, S10, X10 and XS10 groups. The serum was isolated by centrifugation. The concentrations of cytokines (IL-1α, IL-1β, IL-4, TNF-α, IL-12, IFN-γ) were analyzed by multiplex immunoassays using a commercial Luminex immunoassay kit according to the manufacturer’s instructions. Data are shown as mean±s.e.m.; *, *p* < 0.05, **, *p* < 0.01, ***, *p* < 0.001, vs. Sham group; #, *p* < 0.05, ##, *p* < 0.01, ###, *p* < 0.001, vs. S10 group; $, *p* < 0.05, $$, *p* < 0.01, vs. X10 group.

Cytokine secretion is an important mechanism of regulating immune functions. Here, we analyzed several cytokines related to the proliferation, maturation and activation of B lymphocytes and T lymphocytes in peripheral blood. The data showed that the concentrations of IL-1α, IL-1β and IL-4 were significantly increased at 6 h in the X10 and XS10 groups (*p* < 0.05), but IL-1α and IL-4 were rapidly restored to normal level at 7 d after exposure (Figure 3). Importantly, these cytokines were closely associated with the proliferation, maturation, and activation of B lymphocytes, which might contribute to the elevated proportion of B lymphocytes in microwave-exposed rats at 7 d after exposure. 

Moreover, a variety of T-cell immunity cytokines, such as TNF-α, IL-12 and IFN-γ, were also detected at 6 h and 7 d after exposure. At 6 h after exposure, 2.8 GHz microwave irradiation significantly reduced the expression of IL-12 and IFN-γ (*p* < 0.01), while 9.3 GHz microwave irradiation obviously upregulated both TNF-α and IL-12 (*p* < 0.05) but not IFN-γ. Although multifrequency microwaves also downregulated IFN-γ (*p* < 0.05), the regulatory effects were weaker (Figure 3). These data suggested that the T lymphocytes-associated cytokines were differentially regulated by 2.8 GHz and 9.3 GHz microwaves, and further investigations into why will be necessary.

### 3.4. Multifrequency Microwaves Altered the mRNA and Protein Expression Profiles in Peripheral Blood: Transcriptomic and Proteomic Analysis

As described above, multifrequency microwaves obviously caused structural injuries to the immune organs and altered the number, components, and function of the immune cells at 7 d after exposure. To investigate the potential mechanisms, the changes in mRNA and protein expression were evaluated at 7 d after exposure. 

#### 3.4.1. Transcriptomic Analysis

**Figure 4:**
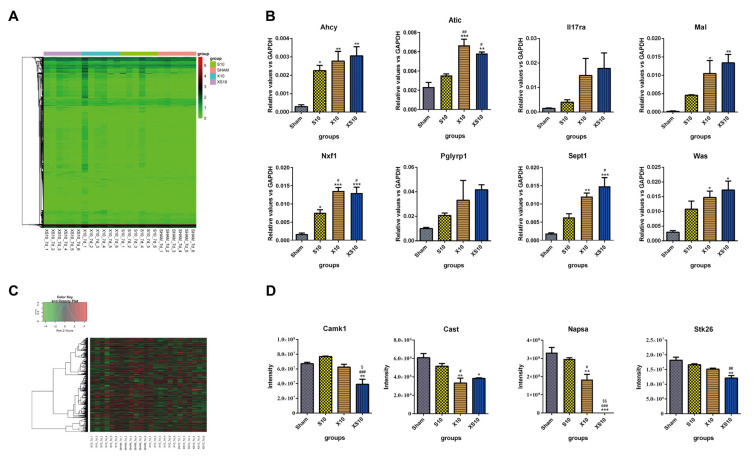
Transcriptomic and proteomic analysis of peripheral blood at 7 d after multifrequency microwave exposure. At 7 d after microwave exposure, peripheral blood was collected from the Sham, S10, X10 and XS10 groups. (**A**,**B**) Transcriptomics analysis. Total RNA was extracted by TRIzol reagent, and RNA sequencing was performed using Illumina NovaSeq. Differential expression analysis between two groups was performed using DESeq2 (v1.20.0) withthe following screening conditions: expression difference multiple |log_2_FoldChange| > 1, significant *q*-value ≤ 0.05. Log2-transformed differentially expressed gene data were used for the expression heatmap by the heatmap1.0.10 package (**A**). The mRNA expression of eight immune activity-associated DEGs was verified by quantitative reverse transcription polymerase chain reaction (qRT-PCR) (**B**). (**C**,**D**) Proteomics analysis. The proteins were extracted from the sera of peripheral blood. After enzymatic degradation and desalination, spectral library was constructed by using a high pH reversed-phase fractionator in data dependent acquisition (DAA) mode. The differentially expressed proteins (DEPs) were analyzed by data independent acquisition (DIA) mode (**C**). The protein expression levels of four DEPs were verified by mass spectrometry (**D**). Data are shown as mean±s.e.m.; *, *p* < 0.05, **, *p* < 0.01, ***, *p* < 0.001 vs. Sham group; #, *p* < 0.05, ##, *p* < 0.01, ###, *p* < 0.001 vs. S10 group; $, *p* < 0.05, $$, *p* < 0.01 vs. X10 group.

Generally, the transcriptomics results showed that microwave irradiation induced differential mRNA expression of numerous genes. Compared to the mRNA expression in the Sham group, 114 upregulated genes and 32 downregulated genes were detected in the S10 group. Many more differentially expressed genes (DEGs) were found in the X10 group, including 373 upregulated genes and 254 downregulated genes. Compared to the S10 and X10 microwaves, multifrequency microwaves induced most DEGs, including 548 upregulated genes and 311 downregulated genes (Figure 4A, Figure S2A and Table 1). Cumulative effects could be induced by multifrequency microwaves. Then, DEGs closely related to immune regulation were selected for verification. Both DEGs directly involved in the immune response (such as *Il17ra*, *Pglyrp1*, *Was* and *Atic*), and protein binding- and protein transport-associated DEGs that indirectly regulate immune responses (such as *Sept1*, *Mal*, *Ahcy* and *Nxf1*) were analyzed by qRT-PCR. The data showed that the expressional alterations of these DEGs were consistent with the results of the transcriptomics (Figure 4B). 

To analyze the potential functions of the DEGs, bioinformatic analysis was conducted. The GO analysis showed that the DEGs were closely associated with cell metabolism and immune regulation. For example, the DEGs were enriched in the biological processes of immune system process, response to stimulus, biological regulation, cellular process, and metabolic process (Figure S3). Moreover, KEGG analysis suggested that the DEGs were enriched in several immune-associated signaling pathways, such as immune-related signaling pathways including the B cell-receptor signaling pathway, endocytosis, the toll-like receptor signaling pathway, antigen processing and presentation, human T-cell leukemia virus 1 infection, the IL-17 signaling pathway, Th17-cell differentiation, leukocyte transendothelial migration, and primary immunodeficiency (Figure S4).

#### 3.4.2. Proteomic Analysis

Compared to mRNA expression, the differentially expressed proteins (DEPs) between the microwave-exposed groups and the Sham group were much lower. In the S10 group, 20 proteins were upregulated and 69 proteins were downregulated, while 16 upregulated proteins and 80 downregulated proteins were found in the X10 group. Moreover, multifrequency microwaves induced more DEPs than single-frequency microwaves, including 25 upregulated proteins and 99 downregulated proteins (Figure 4C, Figure S2C and Table 1). Four DEPs, Camk1, Cast, Napsa, and Stk26, were selected for verification. The mass spectrum results were consistent with the proteomics results (Figure 4D). 

Similar to the results of transcriptomic analysis, the results of the GO analysis showed that many DEPs were enriched in immune-associated biological processes, including the immune response, complement activation (classical pathway), chemokine-mediated signaling pathway, B-cell receptor signaling pathway, positive regulation of B-cell activation, immunoglobulin production (Figure S5). The KEGG analysis suggested that numerous DEPs were pivotal proteins located in immune-related signaling pathways, for example, the autophagy, endocytosis, protein processing in endoplasmic reticulum, calcium signaling pathway, Fc epsilon RI signaling pathway, protein export, mitophagy (Figure S6). 

Combining the transcriptomics and proteomics analyses, we speculated that multifrequency microwaves could regulate the immune balance in the periphery by regulating the mRNA and protein expression of numerous immune-associated genes.

### 3.5. Multifrequency Microwaves Affected the mRNA and Protein Expression Profiles in the Spleen: Transcriptomic and Proteomic Analysis

#### 3.5.1. Transcriptomic Analysis

**Figure 5:**
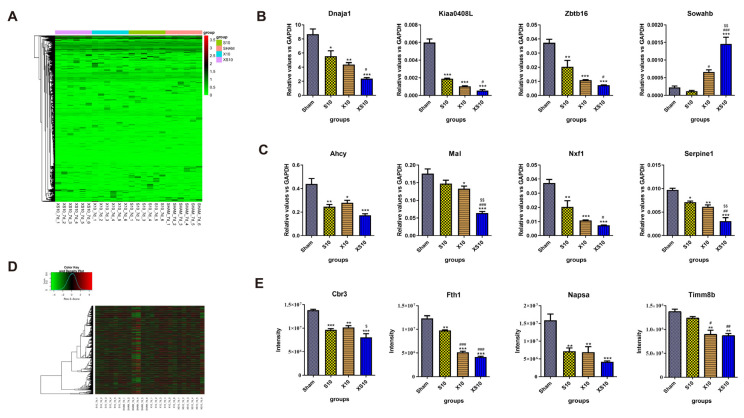
Transcriptomic and proteomic analysis of the spleen at 7 d after multifrequency microwave exposure. At 7 d after microwave exposure, the rats were euthanized, and the spleens were removed from the Sham, S10, X10 and XS10 groups. (**A–C**) Transcriptomics analysis. Total RNA was extracted by TRIzol reagent and RNA sequencing was performed using Illumina NovaSeq, and the DEGs were analyzed as described above (**A**). The mRNA expression levels of four DEGs were verified by qRT-PCR (**B**), and four other immune activity-associated genes were also measured by qRT-PCR (**C**). (**D**,**E**) Proteomics analysis. The proteins were extracted from the spleens. After the spectral library was constructed, the DEPs were analyzed by DDA mode (**D**). The protein expression of four DEPs was verified by mass spectrometry (**E**). Data are shown as mean±s.e.m.; *, *p* < 0.05, **, *p* < 0.01, ***, *p* < 0.001 vs. Sham group; #, *p* < 0.05, ##, *p* < 0.01, ###, *p* < 0.001 vs. S10 group; $, *p* < 0.05, $$, *p* < 0.01 vs. X10 group.

The spleen is the largest peripheral immune organ and plays critical roles in both cellular and humoral immunity. Generally, single-frequency microwaves induced many more DEGs in the spleen than in peripheral blood. Inconsistent with the findings in the peripheral blood, the DEGs in the spleen of the XS10 group were only slightly changed relative to those in the S10 and X10 groups. In detail, 455 upregulated genes and 480 downregulated genes, 475 upregulated genes and 477 downregulated genes, and 483 upregulated genes and 506 downregulated were detected in the S10, X10 and XS10 groups, respectively (Figure 5A, Figure S2B and Table 2). The data suggested that the spleen was much more sensitive to S10 microwaves than the peripheral blood. The expression of eight DEGs, including *Kiaa0408L*, *Dnaja1*, *Zbtb16* and *Sowahb* was verified by qRT-PCR, and the results were consistent with the transcriptomic analysis (Figure 5B). Moreover, several immune response-related genes, such as *Serpine1, Mal, Ahcy* and *Nxf1,* were also detected. Multifrequency microwaves significantly reduced their expression in the spleen, similar to that in peripheral blood (Figure 5C). 

GO analysis showed that the DEGs were mainly enriched in biological processes closely related to immune activity, such as the immune system response, response to stimulus cellular process, cellular component organismal process, multicellular organismal process (Figure S7). KEGG analysis also suggested that the DEGs participated in several immune-related signaling pathways, including the TGF-β signaling pathway, human cytomegalovirus infection, human papilloma virus infection, antigen processing and presentation, and Hippo signaling pathway (Figure S8). These data indicated that microwave exposure could alter the mRNA expression profile to affect the immune homeostasis of the spleen.

#### 3.5.2. Proteomic Analysis

The expression profiles were also analyzed at the protein level by proteomics. Compared to the Sham group, 28 upregulated proteins and 39 downregulated proteins, 79 upregulated proteins and 65 downregulated proteins, and 39 upregulated proteins and 43 downregulated proteins could be detected in the S10, X10 and XS10 groups respectively (Figure 5D, Figure S2D and Table 2). Interestingly, the X10 microwave treatment but not the XS10 microwave treatment, induced the most DEPs among the microwave-exposed groups, which suggested that the S10 and X10 microwave treatment might conversely regulate protein expression in the spleen. Then, four DEPs, including Timm8b, Cbr3, Fth1, and Napsa, were verified by mass spectrometry, and the results were consistent with the proteomic analysis (Figure 5E). 

The above results further demonstrated that microwaves might affect the immune process of the spleen through the regulation of cellular metabolism-related proteins after microwave exposure, given that the regulation of direct immune-related signaling pathways was weak. 

The results of GO analysis showed that DEPs were enriched in metabolism- and immune-associated biological processes, such as glucose metabolic process, negative regulation of B-cell apoptotic process and negative regulation of T-cell energy. Moreover, the DEPs in the X10 groups participated in many immune-related biological processes, such as complement activation, positive regulation of interleukin-10 biosynthetic process, humoral immune response mediated by circulating immunoglobulin, negative regulation of thrombin-activated receptor signaling pathway, positive regulation of interleukin-13 biosynthetic process, positive regulation of immune response to tumor cell, positive regulation of interleukin-4 biosynthetic process and negative regulation of natural killer cell differentiation involved in immune response (Figure S9). KEGG analysis also suggested that the DEPs mainly participated in metabolism and immune-related signaling pathways, such as glutamatergic synapse, glycerophospholipid metabolism, cholesterol metabolism and cysteine and methionine metabolism, TGF-β signaling pathway, and toll-like receptor signaling pathway (Figure S10). These data suggested that microwaves might modulate immune functions in the spleen by both directly affecting immune-associated proteins and indirectly regulating immune cells by influencing cellular metabolism.

## 4. Discussion

Recently, anxiety and concern about the potential health hazards induced by microwaves have been rising with the wide application of microwave technologies. Electromagnetic field radiation, including microwaves, has been classified as a new form of environment pollution that is “possibly carcinogenic to humans” [20]. Until now, most of the studies have focused on the biological effects and potential mechanisms induced by single-frequency microwaves at the indicated average power density [2,3,21,22]. However, people are commonly exposed to complex environments with different types of microwaves in daily life. Therefore, it is interesting to investigate the biological effects induced by microwaves with different frequencies. 

Our group focused on the accumulative effects induced by microwaves with different frequencies. We found that combined 2.8 GHz and 1.5 GHz microwaves impaired spatial memory much more strongly than single-frequency microwaves [18]. Exposure to multifrequency microwaves of 1.5 GHz and 4.3 GHz produced much more serious cognitive impairment in rats [17]. Moreover, the accumulative effects of 1.5 GHz and 2.8 GHz microwaves on cognitive functions were also reported in Wistar rats [23]. S- and X- band microwaves, ranging from 2.0 GHz to 4.0 GHz and 8.0 GHz to 12.0 GHz, are frequently used in daily life, such as in communications. In this study, we found that exposure to multifrequency microwave in the S band (2.8 GHz) and X band (9.3 GHz) produced accumulative injuries to immune organs, including the thymus and spleen. Interestingly, B lymphocytes were active after exposure, which might be attributed to compensatory mechanisms. 

As the first line of defense against external and internal stimuli, the immune system plays an important role in maintaining the body’s homeostasis. It is well-known that microwaves can activate immune responses via both thermal and nonthermal effects. As an adjuvant approach, microwave ablation has been widely used in cancer therapy, such as non-small cell lung cancer, hepatic carcinoma, and colorectal cancer [24,25,26,27]. It has been reported that IL-2 and IFN-γ in plasma increase significantly in patients with non-small cell lung cancer, 1 month after microwave ablation [28]. Moreover, microwave ablation could activate T lymphocyte expansion, induce CD4^+^ T effector and memory cells and increase serum IFN-γ, which shifts the Th1/Th2 balance toward Th1 [11,29]. In metastatic breast cancer models, microwave ablation of the primary tumor inhibited tumor metastasis by activating natural killer (NK) cells via the macrophage/IL-15/NK-cell axis [11]. The abovementioned immune activation induced by microwave ablation could be mainly attributed to thermal effects. However, the nonthermal effects of microwaves on the immune system have always been ignored. Studies from our group and others have investigated the nonthermal effects of microwaves on immune cells both in vitro and in animal models [30,31,32]. In this study, we showed that multifrequency microwaves improved cytokine expression at 6 h after exposure, especially cytokines associated with the proliferation, maturation and activation of B lymphocytes, and then activated B lymphocytes at 7 d after exposure.

To investigate the potential mechanisms, we analyzed the mRNA and protein expression profiles in peripheral blood by transcriptomics and proteomics. Generally, upregulated and downregulated genes, which participate in immune system processes, were detected at the mRNA level in peripheral blood at 7 d after exposure. We found that six B-cell activation-associated genes, *Cxcr5*, *Blnk*, *Pik3cd*, *Lat2*, *Lax1* and *Txlna*, were upregulated after exposure to XS10 microwave irradiation. The CXCL13-CXCR5 axis plays important roles in regulating the motility and trafficking of lymphocytes as well as in activating B lymphocytes [33,34]. B-cell linker protein (BLNK), an essential component of the B-cell antigen receptor signaling pathway, is pivotal for both B-cell development and lymphocyte homeostasis [35,36]. Pik3cd is a key component of the PI3K signaling pathway and is closely associated with B-cell differentiation and maturation [37]. Consistent with the transcriptomics results, numerous genes involved in the biological processes of positive regulation of B-cell activation and B-cell receptor signaling pathway were upregulated. Therefore, we speculated that the alteration of B-cell activation-related genes might contribute to the increase in B lymphocytes in peripheral blood at 7 d after exposure to XS10 microwaves. Moreover, at the protein level, XS10 microwaves also upregulated nine proteins related with T-cell activation, including Ada, Treml2, Cd4, Hsh2d, Nlrc3, Was and Vav1, suggesting that T lymphocytes, especially CD4^+^ T lymphocytes were also activated at 7 d after exposure. Although multifrequency microwaves caused reversible injuries to the thymus and spleen, the functions of immune cells in the peripheral blood were activated, which might initiate the recovery of immune organs.

The spleen, the largest organ in the human immune system, can produce certain types of immune cells, such as B and T lymphocytes, and it plays important roles in immune regulation. We also analyzed mRNA and protein expression profiles in the spleen at 7 d after exposure. In addition to immune activation associated DEGs and DEPs, numerous DEGs and DEPs were enriched in signaling transduction, DNA duplication, and cellular metabolism-related biological processes, such as negative regulation of DNA endoreduplication, regulation of signal transduction, and glutathione catabolic process. Therefore, we hypothesized that multifrequency microwaves of 2.8 GHz and 9.3 GHz could affect DNA duplication, cellular metabolism and related signaling transduction in the spleen, which in turn activated immune cells and triggered their migration into the peripheral blood. Identification of DEGs and DEPs has been emerged as an effective approach for clarifying the mechanisms underlying certain biological effect. In this study, we analyzed DEGs and DEPs at 7 d after exposure, which is an ideal time point to analyze the acute responses of immune system to microwave exposure. However, the potential mechanisms that might cause long-term biological effects might be neglected. In the future, we are interested to explore the recovery mechanism via analyzing DEGs and DEPs at the recovery stage after microwave exposure.

In our daily life and working environment, people always long-term exposed to microwaves, such as mobile phone, radar, and medical equipment. Recent years, the biological effects induced by long-term exposure to microwave has been attracting more and more attentions. Our group and others reported that long-term exposure to S band, L band or 900 MHz microwave caused significant pathological injuries in brain and impaired the spatial learning and memory ability [2,38,39,40,41]. Studies also reported that 900 MHz microwave, a classical mobile phone radiation, could disrupt the integrity of blood-brain barrier in rats [42]. In addition to the nervous system, long-term exposure to 2.45-GHz microwave could adversely affect the reproductive function in male mice [43]. Moreover, numerous studies focused on the carcinogenic effects induced by long-term exposure to low intensity microwave, such as mobile phone radiation. Some epidemiological studies suggested that long-term exposure to low-intensity microwaves can promote cancer development [44,45]. In tumor-prone and intact rodent models, significant increases in carcinogenesis could be observed after 17–24 months’ microwave exposure. However, others reported negative results after long-term exposure [46,47]. The immune system plays pivotal roles in surveilling tumor initiation and progression. It has been reported that two weeks’ exposure to 915 MHz microwave induced DNA damage of leukocytes in peripheral blood, which might cause by oxidative stress [48]. Therefore, it will be interesting to explore the biological effects and potential mechanisms after long-term exposure to multifrequency microwave of 2.8 GHz and 9.3 GHz.

In conclusion, we found that multifrequency microwaves of 2.8 GHz and 9.3 GHz caused obvious injuries to both the thymus and spleen at 7 d after exposure, which then recovered by 28 d after radiation. Unexpectedly, multifrequency microwave exposure increased the immune cells in the periphery, especially B lymphocytes, which might be attributed to elevated cytokines, as well as upregulation of numerous immune-associated genes, such as *Cxcr5*, *Blnk* and *Pik3cd*. Moreover, the transcriptomic and proteomic analysis of peripheral blood and spleen suggested that alterations of DNA duplication, cellular metabolism and signal transduction might be involved in microwave-induced immune activation. 

## Figures and Tables

**Table 1:** The number of differentially expressed genes and proteins in the peripheral blood after exposure to multifrequency microwaves of 2.8 GHz and 9.3 GHz.

Groups	Number of DEGs		Number of DEPs	
	Upregulated	Downregulated	Upregulated	Downregulated
S10 vs. Sham	114	32	20	69
X10 vs. Sham	373	254	16	80
XS10 vs. Sham	548	311	25	99
X10 vs. S10	3	3	14	45
XS10 vs. S10	212	146	32	78
XS10 vs. X10	22	10	24	15

**Table 2:** The number of differentially expressed genes and proteins in the spleen after exposure to multifrequency microwaves of 2.8 GHz and 9.3 GHz.

Groups	Number of DEGs		Number of DEPs	
	Upregulated	Downregulated	Upregulated	Downregulated
S10 vs. Sham	455	480	28	39
X10 vs. Sham	475	477	79	65
XS10 vs. Sham	483	506	39	43
X10 vs. S10	465	485	66	41
XS10 vs. S10	467	451	36	19
XS10 vs. X10	459	428	23	25

## Data Availability

Not applicable.

## Supplementary Materials

The following are available online at https://www.mdpi.com/article/10.3390/cells11233849/s1, Figure S1: The histopathological analysis of bone marrow in rats after exposure to multifrequency microwave of 2.8 GHz and 9.3 GHz; Figure S2: The heatmap of differential expressed genes (DEGs) and different expression prtoeins (DEPs) at 7 d after microwave exposure; Figure S3: Gene Ontology (GO) enrichment of differentially expressed genes (DEGs) in peripheral blood; Figure S4: Kyoto Encyclopedia of Genes and Genomes (KEGG) pathway enrichment of DEGs in peripheral blood; Figure S5: Gene Ontology (GO) enrichment of differentially expressed proteins (DEPs) in peripheral blood; Figure S6: Kyoto Encyclopedia of Genes and Genomes (KEGG) pathway enrichment of DEPs in peripheral blood; Figure S7: Gene Ontology (GO) enrichment of DEGs in spleen; Figure S8: Kyoto Encyclopedia of Genes and Genomes (KEGG) pathway enrichment of DEGs in spleen; Figure S9: Gene Ontology (GO) enrichment of DEPs in spleen; Figure S10: Kyoto Encyclopedia of Genes and Genomes (KEGG) pathway enrichment of DEPs in spleen. Table S1: Quantitative PCR primer sequences.
