# Supplementary material for: The Biological Effects of Compound Microwave Exposure with 2.8 GHz and 9.3 GHz on Immune System: Transcriptomic and Proteomic Analysis
Source: Cells. 2022 Nov 30;11(23):3849. doi: 10.3390/cells11233849 (PMC9735949; doi:10.3390/cells11233849)
Supplement: Supplementary file 1 [file cells-11-03849-s001.zip › Supplementary Table ST1.pdf]

Supplementary Table S1 Quantitative PCR primer sequences

Gene name	Forward (F) and Reverse (R) primer sequences
Ahcy	F：TCCAAACTCTCGCTTGGTCT
	R：AGCCTTCAAACTCCAGTGGT
Atic	F：TTGGTGAGGGTGAAGACCTG
	R：AGCATCGGAACTGACAGACA
Il17ra	F：CTCTGGGTGTATGGCCTCAT
	R：TGATCAGCACCAGAAAGCCT
Mal	F：GCAGTGGTGTTCGCCTATG
	R：TGGGTTTCAGCTCCCAATCT
Nxf1	F：CTCTCCCAAGAACAGCAGGA
	R：TCTCCCTTGGCCTTGAGATG
Pglyrp1	F：GTGACTACTCACACCGGGTA
	R：TGGTCCCAGCTTTGGATGAT
Sept1	F：CAAGCTTTCCAGGCAGAGTG
	R：GCTTGCATCTTCTCCAGCAT
Was	F：GAGAACTCAGTACCGCAAGC
	R：CCCTTCATCTGAGGAGTGGA
Cpeb4	F：CCCGTTACGTTCCAAAGGTC
	R：GCTGCTTCGCTGTTCTGTTA
Dnaja1	F：AGGACTGGAGCCAGGAGATA
	R：TCTGACCTGGATGAGAGGTG
Kiaa0408L	F：GCCCTTAACACCGCTCTTG
	R：CTGGCCATTGCTGATCAAGT
Sowahb	F：TTGCGACTGGAGAAGGTAGG
	R：ATGGCAATGACACCCAGAGA
Zbtb16	F：GTTCACACAGGTGAGAAGCC
	R：ATGGTGCATTGGTAGGGTGA
GAPDH	F：CAAGGCTGAGAATGGGAAGC
	R：GAAGACGCCAGTAGACTCCA
