# Supplementary figures and images for: The Biological Effects of Compound Microwave Exposure with 2.8 GHz and 9.3 GHz on Immune System: Transcriptomic and Proteomic Analysis
Source: Cells. 2022 Nov 30;11(23):3849. doi: 10.3390/cells11233849 (PMC9735949; doi:10.3390/cells11233849)

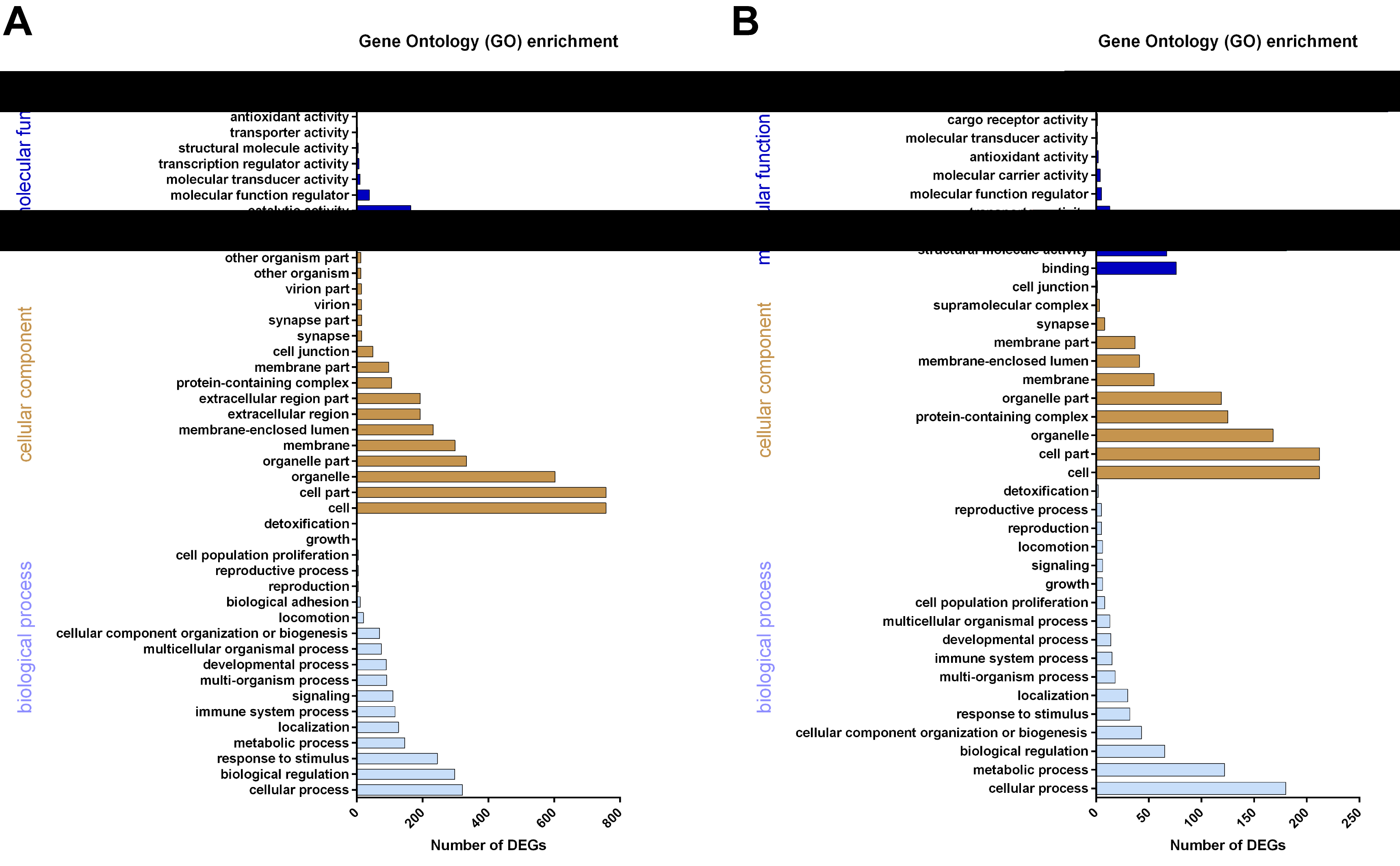

Supplement: Supplementary file 1 [file cells-11-03849-s001.zip › Supplementary Figure SF1.tif]

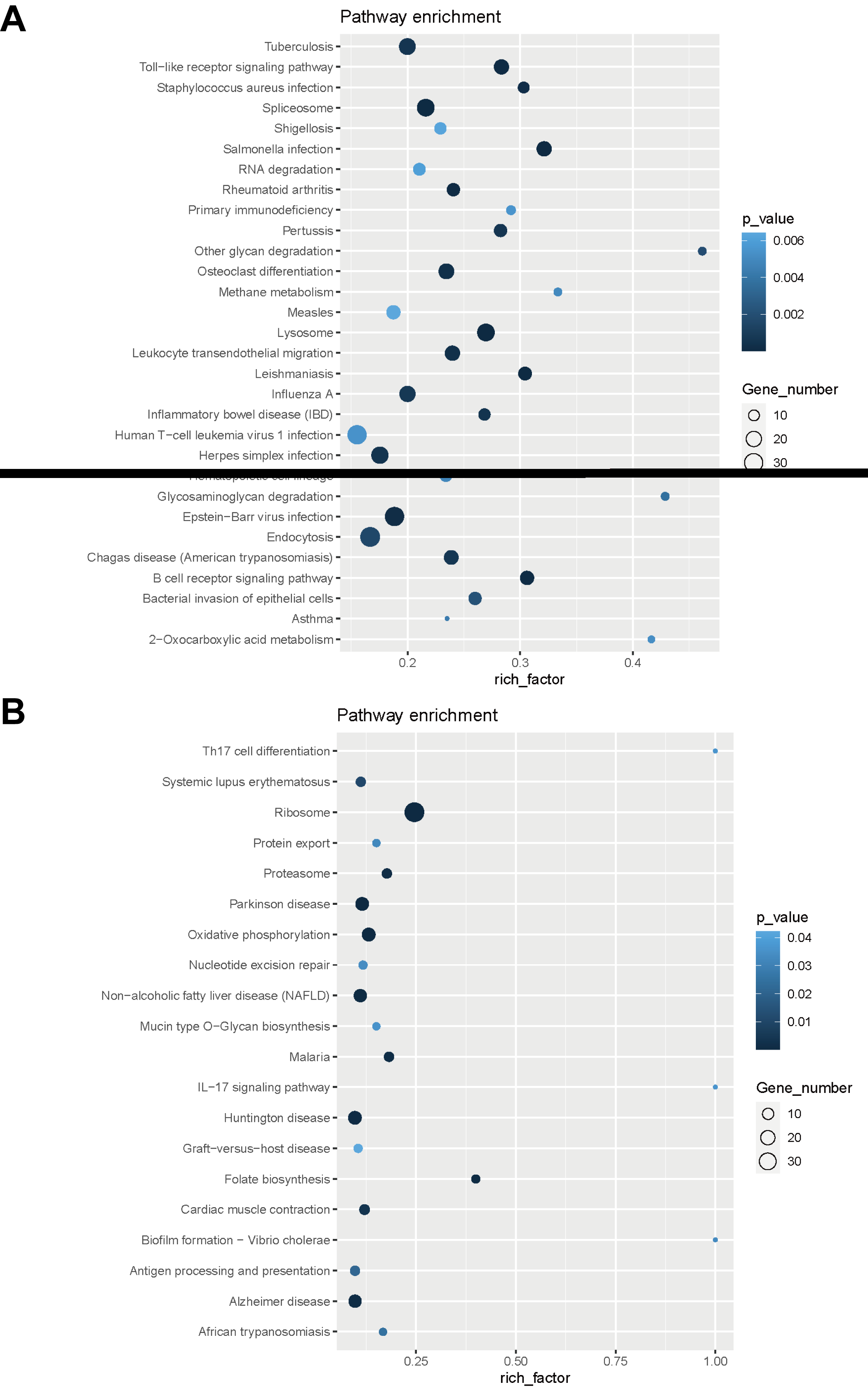

Supplement: Supplementary file 1 [file cells-11-03849-s001.zip › Supplementary Figure SF2.tif]

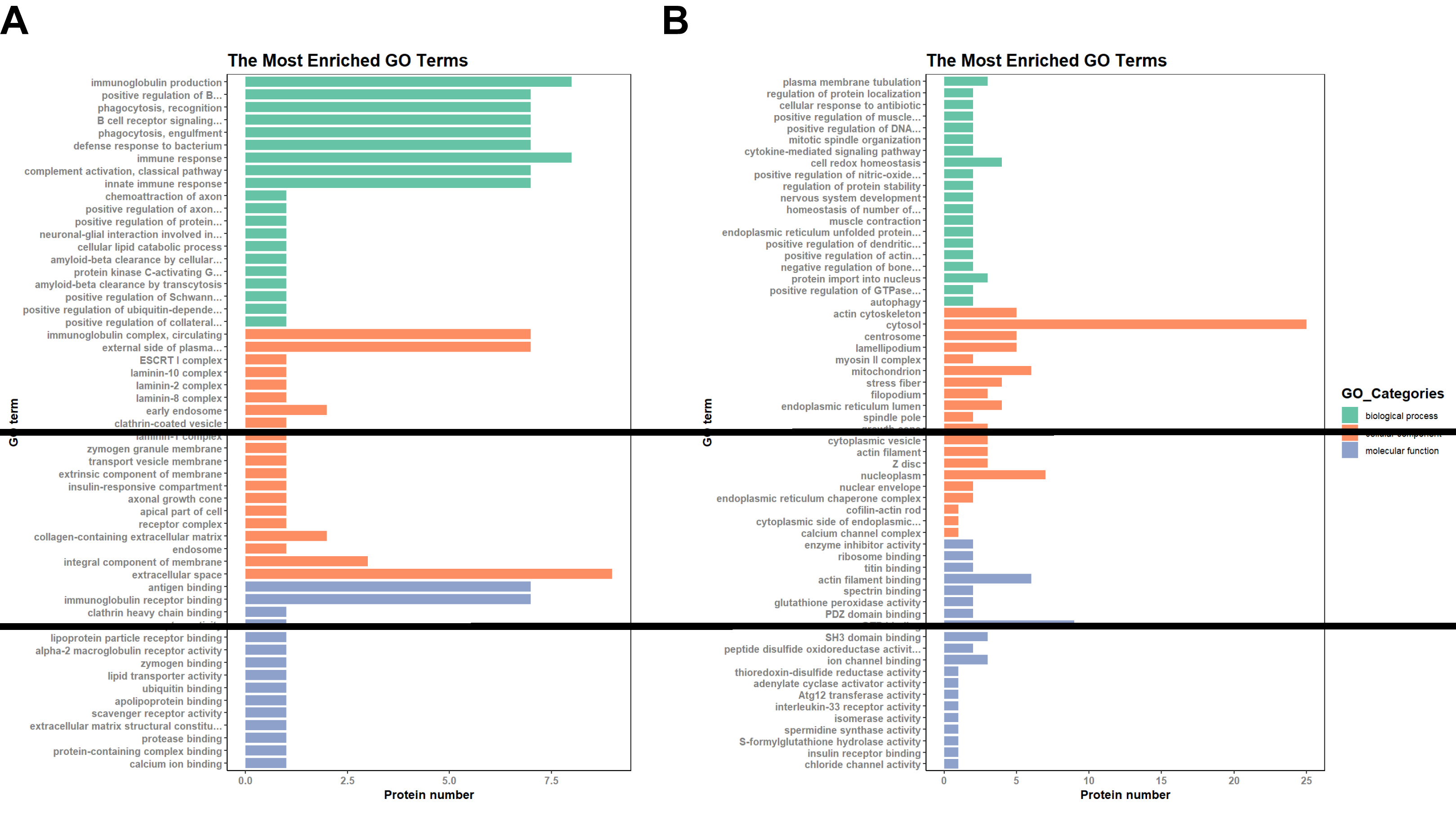

Supplement: Supplementary file 1 [file cells-11-03849-s001.zip › Supplementary Figure SF3.tif]

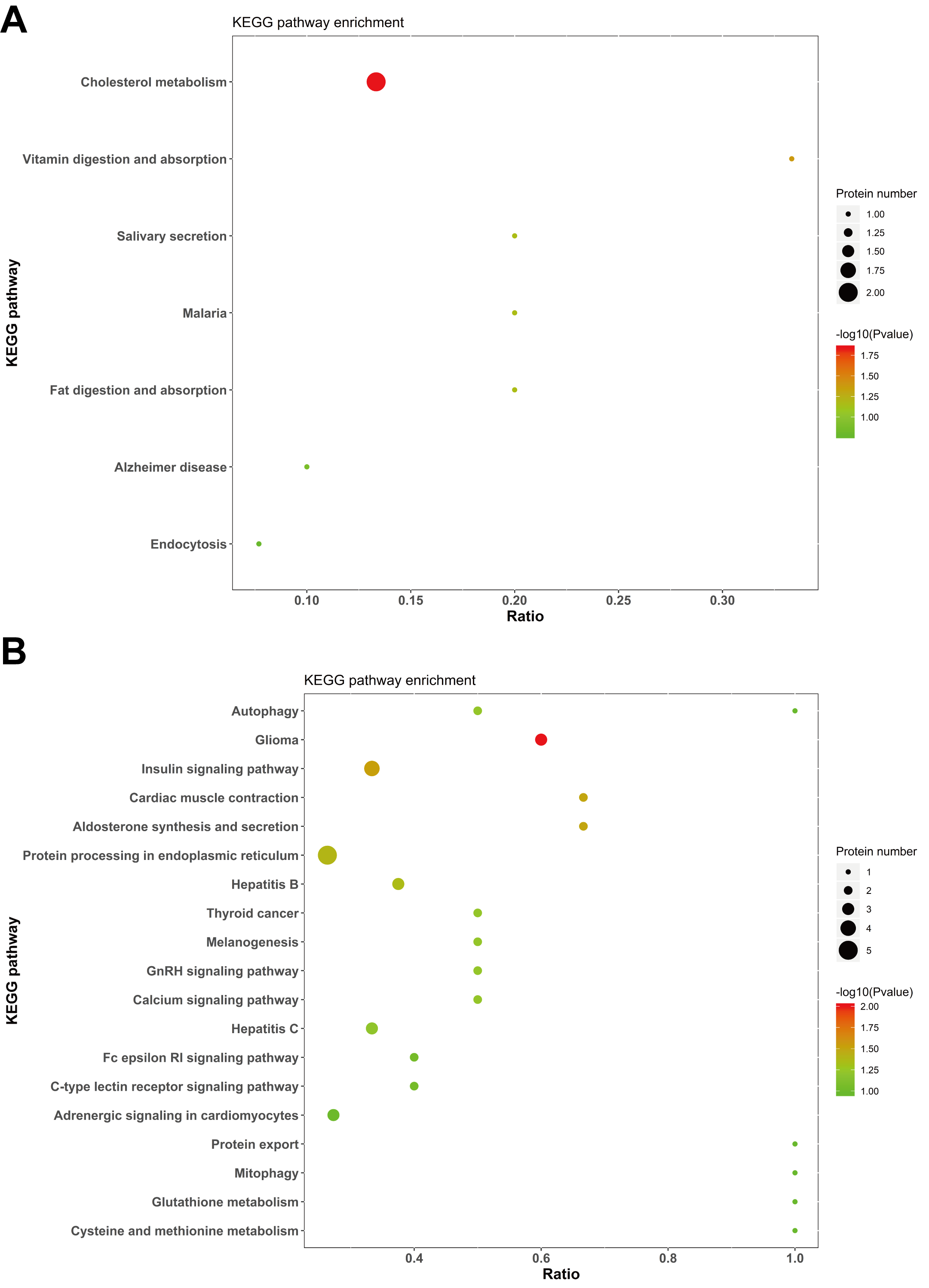

Supplement: Supplementary file 1 [file cells-11-03849-s001.zip › Supplementary Figure SF4.tif]

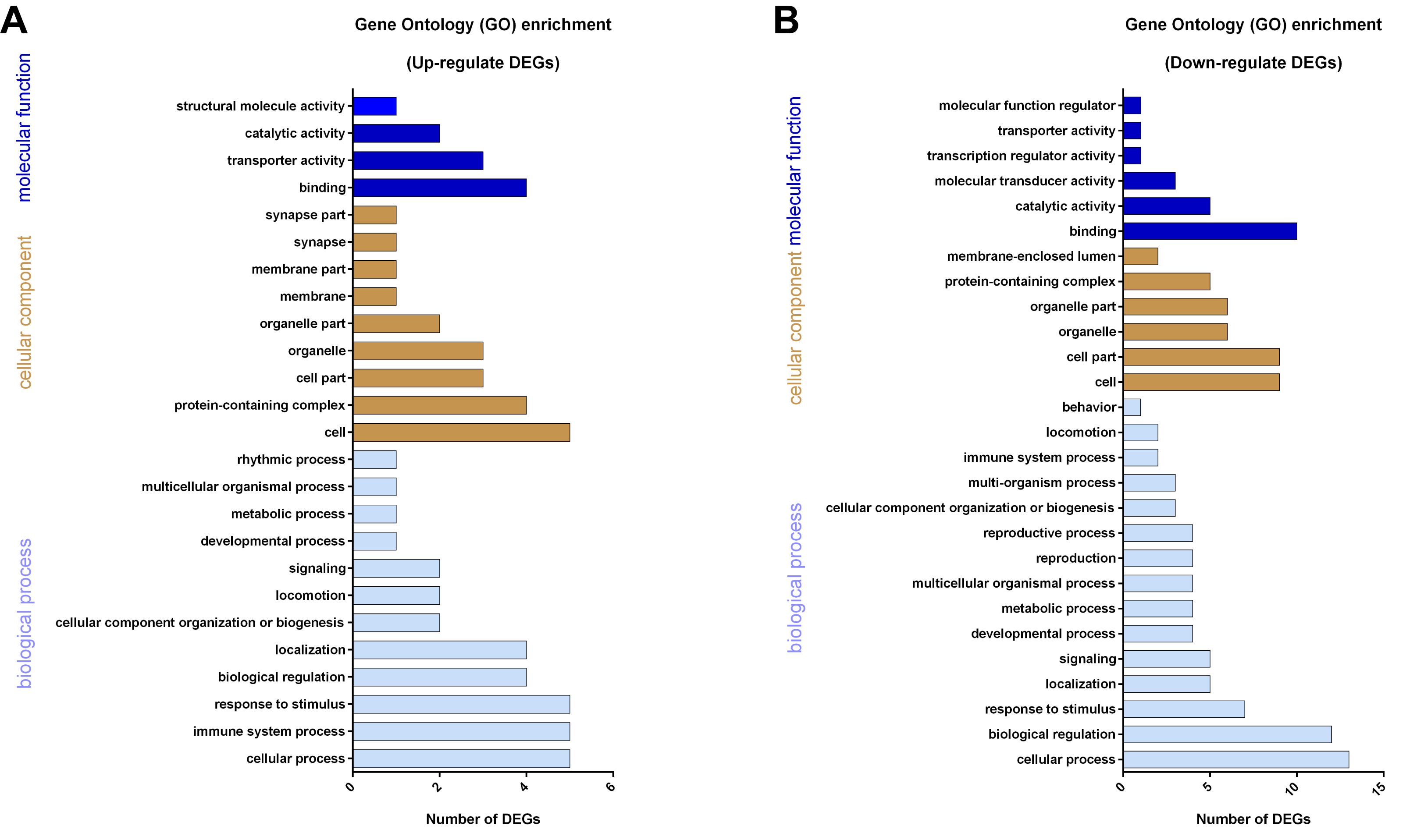

Supplement: Supplementary file 1 [file cells-11-03849-s001.zip › Supplementary Figure SF5.tif]

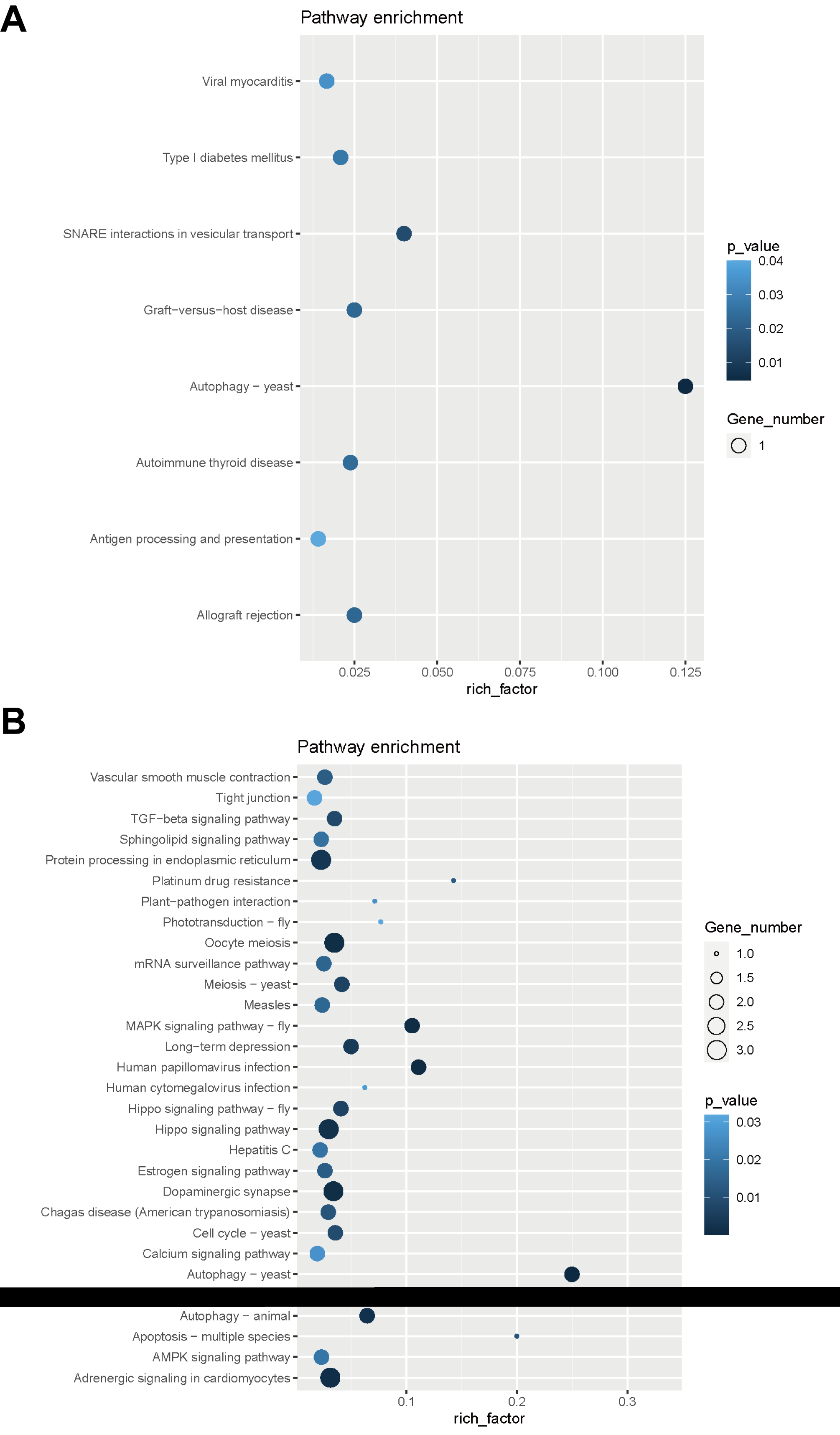

Supplement: Supplementary file 1 [file cells-11-03849-s001.zip › Supplementary Figure SF6.tif]

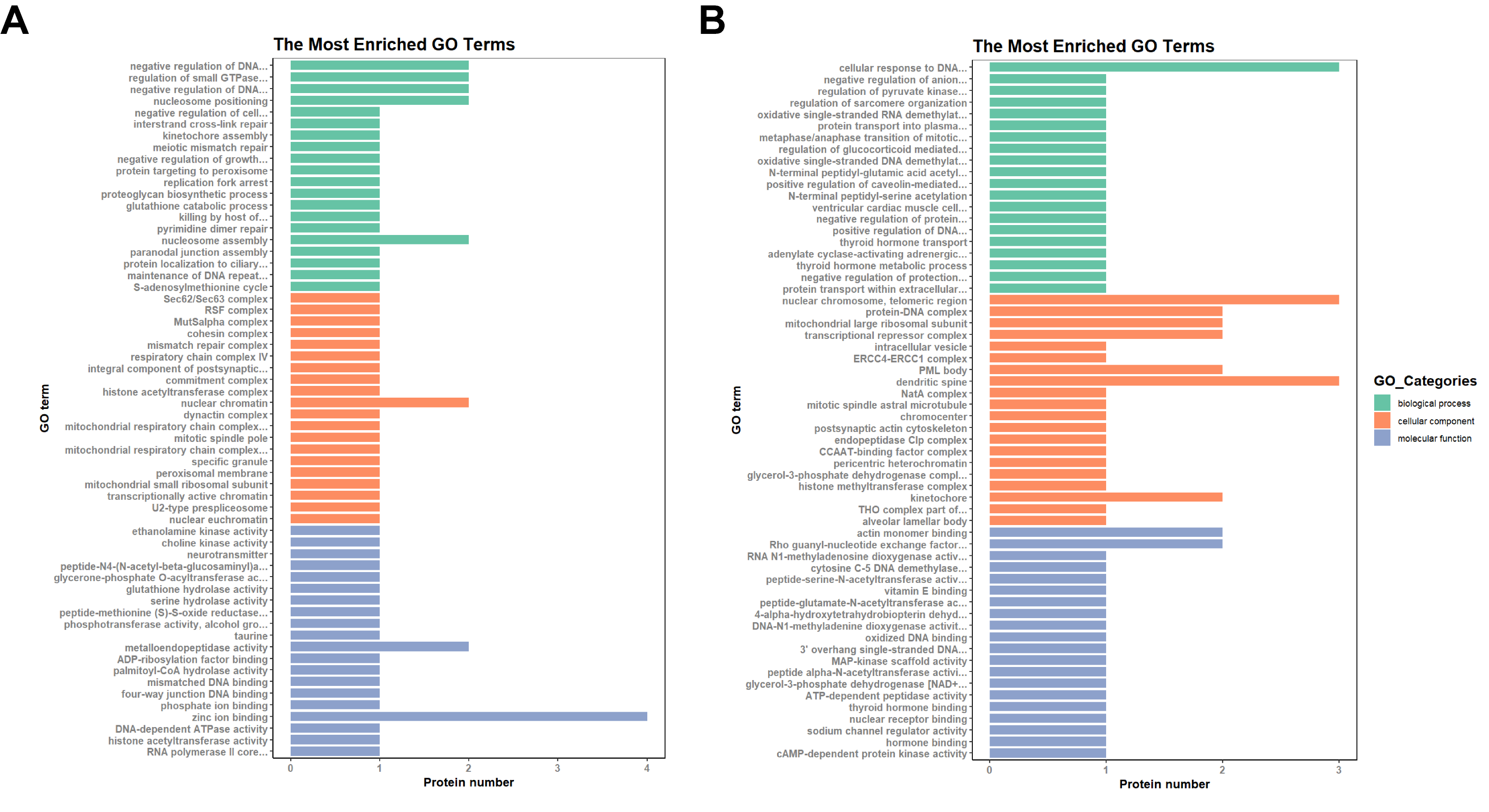

Supplement: Supplementary file 1 [file cells-11-03849-s001.zip › Supplementary Figure SF7.tif]

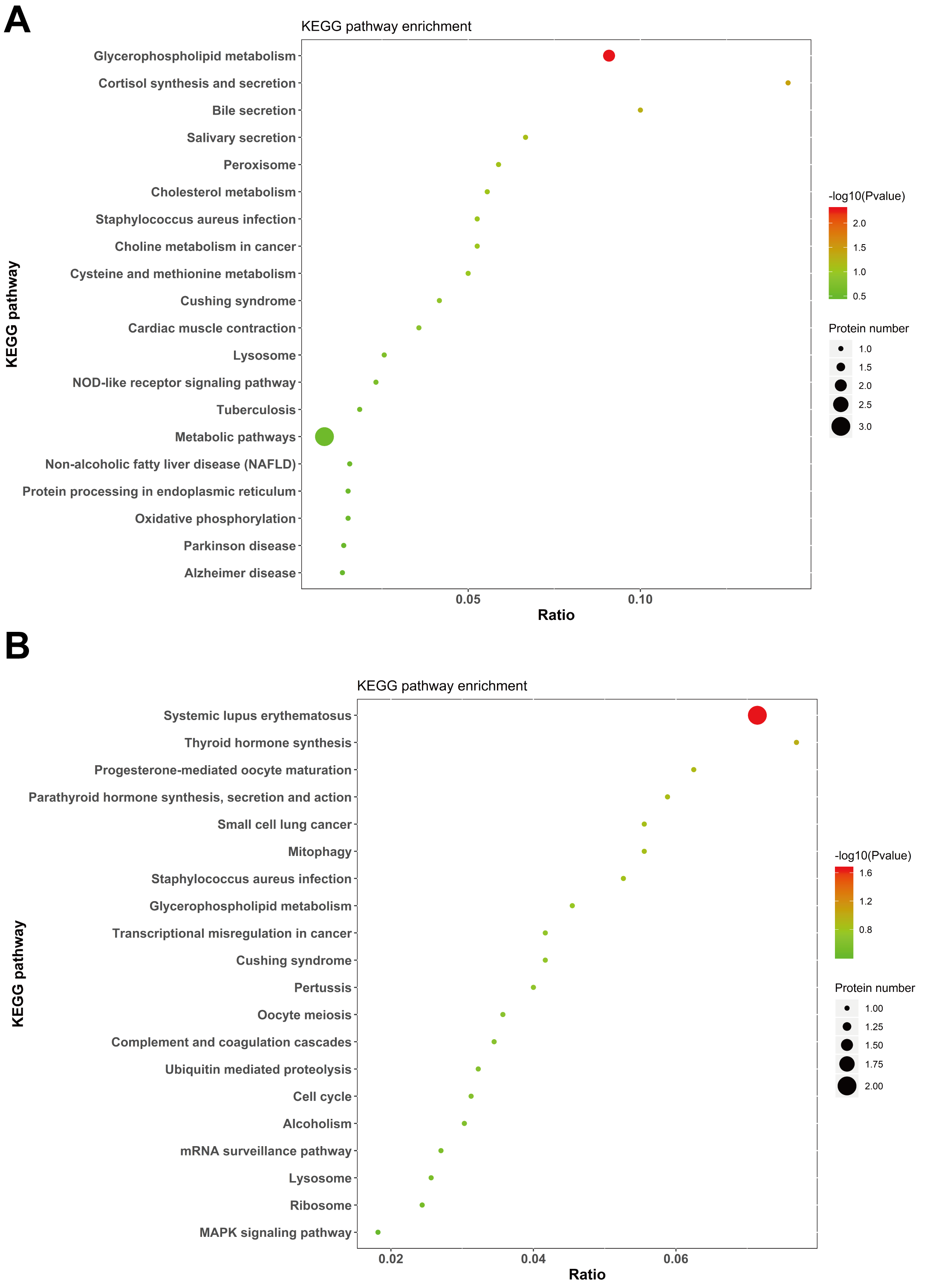

Supplement: Supplementary file 1 [file cells-11-03849-s001.zip › Supplementary Figure SF8.tif]
